# Can Thymidine Phosphorylase Be a Predictive Marker for Gemcitabine and Doxifluridine Combination Chemotherapy in Cholangiocarcinoma? Case Series

**DOI:** 10.1097/MD.0000000000000305

**Published:** 2014-12-02

**Authors:** Myoung Hee Kang, Won Sup Lee, Se-Il Go, Moon Jin Kim, Un Seok Lee, Hye Jung Choi, Dong Chul Kim, Jeong-Hee Lee, Hoon-Gu Kim, Kyung Soo Bae, Jae Min Cho

**Affiliations:** From the Department of Internal Medicine (MHK, WSL, S-IG, MJK, USL, HJC, H-GK); Department of Pathology (DCK, J-HL); and Department of Surgery, Institute of Health Sciences, Gyeongsang National University School of Medicine, Jinju, South Korea (KSB, JMC).

## Abstract

Unresectable cholangiocarcinoma is poorly responded to chemotherapy, especially for the case refractory to gemcitabine and cisplatin. Here, we tested whether high expression of thymidine phosphorylase (TP) can be a predictive biomarker for the indicator for gemcitabine and doxifluridine combination chemotherapy in the cholangiocarcinoma refractory to gemcitabine and cisplatin.

Immunohistochemical staining for TP was performed with a biopsy specimen. We accepted the result as positive when more than 10% of cancer cells were stained with moderate intensity.

Here, we report 2 cases of TP-positive cholangiocarcinoma well controlled with gemcitabine and doxifluridine combination chemotherapy, which had been refractory to the first line treatment with gemcitabine and cisplatin combination chemotherapy.

## INTRODUCTION

Cholangiocarcinoma is a tumor originating from bile duct epithelial cells. According to the autopsy results, the worldwide incidence is 0.01% to 0.5%, which indicates that cholangiocarcinoma is a relatively rare tumor.^1^ The incidence appears to vary according to the geographical region; it is higher in Asia than in the western countries. In Korea, it has been reported to be as high as 2.6%.^2^ Although the etiology and carcinogenesis are not fully elucidated, chronic inflammation in the bile duct plays an important role in the development of cholangiocarcinoma. Congenital bile duct deformity, primary sclerosing cholangitis, intrahepatic stone, parasite infection, chronic typhoid carriers, and papillary changes of the bile duct are the risk factors for the development of cholangiocarcinoma.^3,4^ Operable cholangiocarcinoma can be cured by radical surgery, but 5-year survival rate ranges from 10% to 40%. In inoperable cases, the prognosis was dismal, and the overall median duration of survival is less than 6 months.^5,6^

For the palliation of the patients with inoperable cholangiocarcinoma, the gemcitabine-based combination chemotherapy is widely used; gemcitabine and cisplatin combination chemotherapy is one of the most commonly used regimens.^7^ Nonetheless, even in patients who have responded to this treatment, the median survival rate is shorter than 12 months, and thus new treatment methods are required.^8^ For the case refractory to gemcitabine and cisplatin combination chemotherapy, few optional therapeutic trials are available.

5-Fluorouracil (5-FU) is one of the most commonly used chemotherapeutic agents for gastrointestinal cancer. Doxifluridine (5-deoxy-5-fluorouridine), a prodrug of 5-FU, which is converted to 5-FU via thymidine phosphorylase (TP), generates 5-FU preferentially in cancer,^9^ because various cancer tissues have higher levels of TP expression than adjacent non-neoplastic tissue.^10,11^ In addition, phase II trials showed that combination chemotherapy of gemcitabine and capecitabine (which is converted to 5-deoxy-5-fluorouridine in the liver and/or tumor tissue) had response rates ranging 30% to 48%,^12–14^ suggesting that they have synergistic effects. From these results, we assumed that gemcitabine and doxifluridine combination chemotherapy can be effective in cholangiocarcinoma even it is refractory to gemcitabine and cisplatin combination chemotherapy if the cholangiocarcinoma expressed TP highly in tumor cells.

Here, we report the results of 2 cases of TP-positive cholangiocarcinoma well controlled by gemcitabine and doxifluridine combination chemotherapy after they had been refractory to the first line treatment with gemcitabine and cisplatin combination chemotherapy. These results suggest that TP positivity might be a candidate for the predictive marker for the gemcitabine and doxifluridine combination chemotherapy.

## CASE 1

A 62-year-old female patient was admitted for discomfort in the upper abdominal area as the chief complaint. The patient visited at “S” hospital for the symptom and diagnosed as cholangiocarcinoma. Although laparoscopic resection was attempted, surgery could not be performed because of the presence of peritoneal seeding. Then she was treated with 1cycle of gemcitabine and cisplatin combination chemotherapy (gemcitabine 1200 mg/m^2^ Day 1 and 8, cisplatin 60 mg/m^2^ Day 1, every 3 weeks), and transferred to our hospital. At the time of admission, vital signs were within normal ranges. In peripheral blood test, leukocyte was 4920/μL, hemoglobin was 10.0 g/dL, and platelet was 359,000/μL. In serum biochemistry test, they were also within normal ranges. At the time of admission, tumor marker tests were not performed.

### Clinical Courses

**FIGURE 1:**
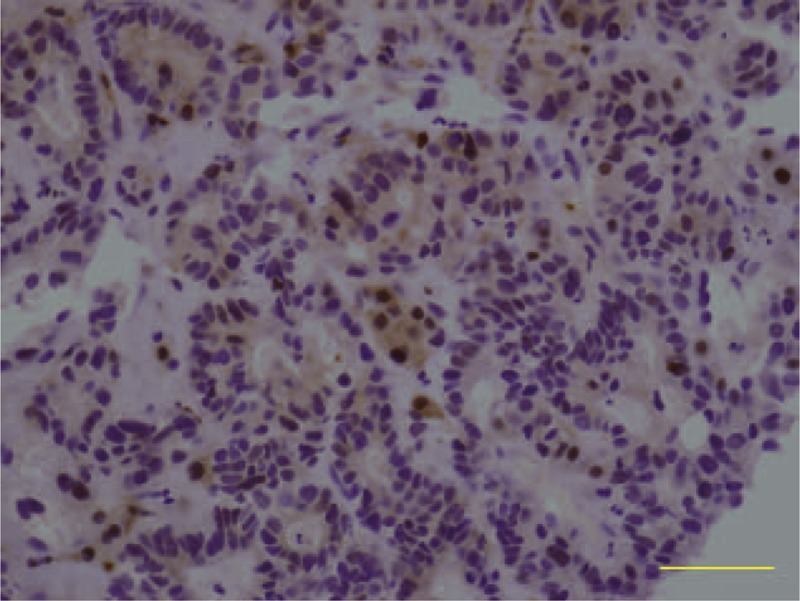
Immunohistochemical staining for TP (400×). Intensive cytoplasmic and nuclear staining is observed in the cancer tissue.

After 2nd cycle of combination chemotherapy with gemcitabine and cisplatin, we performed abdominal computed tomography (CT) for the response evaluation. The abdominal CT revealed that the size of tumor increased as compare to baseline CT findings. For the next therapeutic plan, we performed immunohistochemical staining for TP with a surgical biopsy specimen, and the result, as shown in Figure 1, was positive immunohistochemical staining for TP.

**FIGURE 2:**
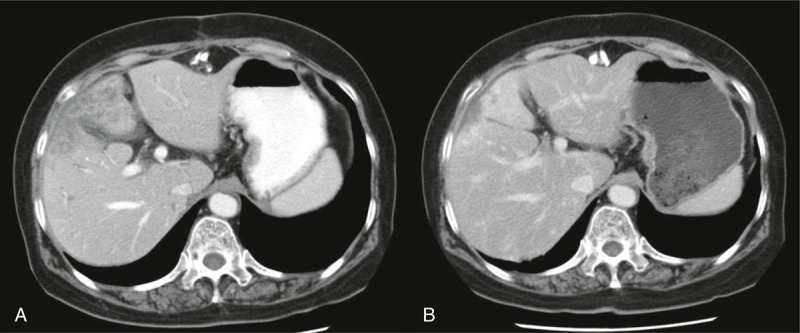
Abdominal CT scan before and after gemcitabine/doxifluridine combination chemotherapy. (A) Baseline CT finding. (B) Three weeks after 4 cycles of the combination chemotherapy.

Afterward, chemotherapeutic agents were switched to gemcitabine and doxifluridine regimen (gemcitabine 1200 mg/m^2^ Day 1 and 8, doxifluridine 1200 mg divided 3 times p.o. daily, every 3 weeks), and administered 4 cycles. The abdominal CT scan revealed that shrinkage of main mass indicating partial response (Figure 2). The patient chose to terminate chemotherapy, and was observed at the outpatient clinic even though the adverse events were minimal. The CT scan performed during the follow-up revealed the progression of disease (remission duration: 9 months), and then combination chemotherapy was restarted with the previous gemcitabine and doxifluridine regimen. The abdominal CT scan performed after 2 cycle of chemotherapy showed the disease progression. Afterward, maintained with doxifluridine alone until the patient's condition was deteriorated and the patient died of sepsis (overall survival: 22 months).

## CASE 2

**FIGURE 3:**
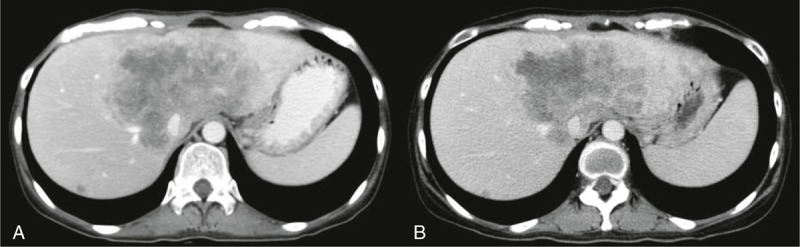
Abdominal CT scan before and after gemcitabine/doxifluridine combination chemotherapy. (A) Baseline CT finding. (B) Three weeks after 4 cycles of the combination chemotherapy.

**FIGURE 4:**
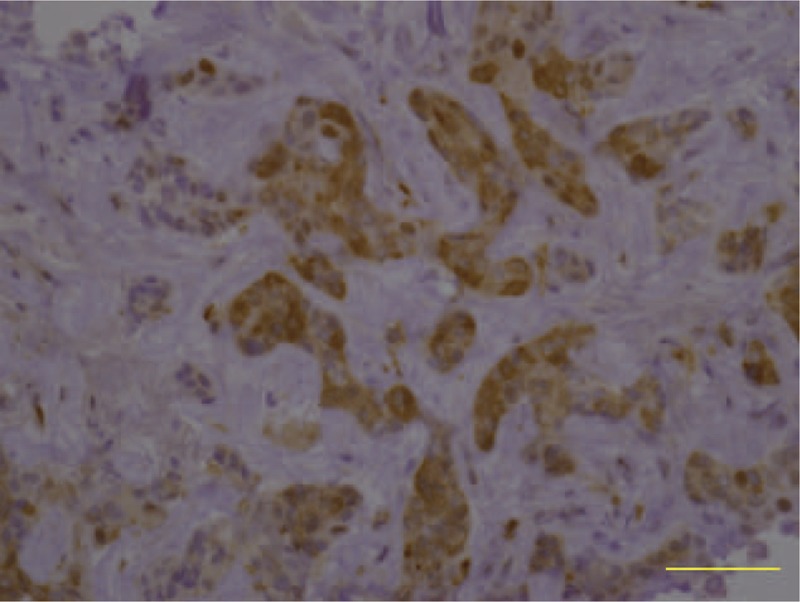
Immunohistochemical staining for TP (400×). Intensive cytoplasmic and nuclear staining is observed in the cancer tissue.

A 32-year-old female patient was admitted for discomfort in the upper abdominal area as the chief complaint. The patient was diagnosed as cholangiocarcinoma. She was transferred to “A” hospital, treated with 4-cycle of gemcitabine and cisplatin combination chemotherapy (gemcitabine 1000 mg/m^2^ Day 1 and 8, cisplatin 60 mg/m^2^ Day 1, every 3 weeks), and then transferred back to our hospital due to disease progression after chemotherapy. On admission, vital signs were within normal ranges. In peripheral blood test, leukocyte was 4927/μL, hemoglobin was 9.1 g/dL, and platelet was reduced to 49,000/μL. In serum biochemistry test, they were within normal ranges except that alkaline phosphatase was mildly elevated to 174 IU/L. Abdominal CT scan revealed 12-cm sized huge mass invading portal vein (Figure 3A). For the next therapeutic plan, immunohistochemical staining for TP was also performed with a biopsy specimen, which revealed positive immunoreactivity to TP (Figure 4).

### Clinical Courses

On admission, gemcitabine and doxifluridine combination chemotherapy (gemcitabine 1000 mg/m^2^ Day 1 and 8, doxifluridine 1200 mg divided 3 times p.o. daily from Day 1 to 14, every 3 weeks) was initiated. For 11 months, the cancer lesion had been controlled as stable disease as shown in CT scan (Figure 3B). The abdominal CT scan performed at 11 months after treatment indicated the disease progression, and thus chemotherapeutic agents were switched to irinotecan and capecitabine combination chemotherapy. Nonetheless, the cancer was not responded to it, and she died due to disease progression (overall survival: 16 months).

## DISCUSSION

Inoperable advanced cholangiocarcinoma is poorly responded to chemotherapy, and the prognosis was dismal with the median survival ranging 6 to 12 months.^7^ Up to now no combination chemotherapy has been proved to prolong the survival of the patients with cholangiocarcinoma. Hence, many clinical trials have been carried out to find a new therapy. In clinical practice, gemcitabine-based chemotherapy has been widely used. However, in the case of cholangiocarcinoma refractory to gemcitabine and cisplatin combination chemotherapy, few therapeutic trials are available. Here, confirming the overexpression of TP in cancer tissue, we used gemcitabine and doxifluridine combination chemotherapy for these refractory cases to gemcitabine and cisplatin combination chemotherapy. We found that this regimen was tolerable and effective even though the effects should need to be evaluated in prospective clinical trials. In phase II trials, it has been reported that combination chemotherapy of gemcitabine and capecitabine had some efficacy against cholangiocarcinoma and its toxicities was mild.^14^ Here, we performed immunohistochemical staining before starting chemotherapy and found that both the case was as strong positive immunoreactivity to TP as the TP expressions in gastric cancer.^15^ We used the same criteria as we used in gastric cancer in the previous report.^15^ The percentage of the positive cell of both case were more than 30%. The high level of TP expression in tumor tissue theoretically bring about tumor-selectively high concentration of 5-FU, which can result in good response to doxifluridine-based combination chemotherapy and minimal toxicities from this regimen. In addition, it has been reported that TP expression can be a predictive marker for oral 5-FU prodrug, which will be converted to 5-FU by TP.^16,17^ The treatment efficacy of doxifluridine was actually proven in gastric cancer, colon cancer, and other digestive tract cancers.^10,11^ These finding supports our therapeutic plan. In the 2 patients, time to progression for this combination chemotherapy was 9 and 11 months and the overall survival was 22 and 16 months, which appear to be longer than the average survival period of inoperable cholangiocarcinoma patients who had been refractory to the 1st-line combination chemotherapy.

Clinical studies showing that the overexpression of TP enhances the treatment efficacy of doxifluridine or capecitabine have not been reported in cholangiocarcinoma, yet. This is the first study suggesting that the gemcitabine and doxifluridine combination chemotherapy should be used as the second-line treatment for palliation in the cholangiocarcinoma showing positive expression for TP. The reason why we used doxifluridine instead of capecitabine is that doxifluridine is covered by medical insurance in Korea.

It is unclear that gemcitabine and doxifluridine therapy can be applied for all the cholangiocarcinoma because not only the frequency of the TP expression but also the efficacy of positive TP expression as predictive marker in cholangiocarcinoma has not been investigated yet. We are under investigation on the frequency of TP expression in cholangiocarcinoma. Further prospective clinical trial is warranted for this regimen to determine whether TP positivity can be a predictive marker for the gemcitabine and doxifluridine combination chemotherapy in cholangiocarcinoma.
